# Supplementary material for: Validation of the Relationships Questionnaire (RQ) against the Experience of Close Relationship-Revised Questionnaire in a Clinical Psychiatric Sample
Source: Healthcare (Basel). 2021 Sep 7;9(9):1174. doi: 10.3390/healthcare9091174 (PMC8470855; doi:10.3390/healthcare9091174)
Supplement: Supplementary file 1 [file healthcare-09-01174-s001.zip › healthcare-1317225-supplementary.pdf]

Table S1. Relationship between IIP subscales and ECR-R scales.

	DO	VI	CO	SI	NO	OA	SS	IN
ECR-R anxiety	0.307**	0.135	0.340**	0.352**	0.255**	0.388**	0.350**	0.313**
ECR-R avoidance	−0.063	0.106	0.191*	0.178*	0.087	0.184*	0.033	−0.031

N=143; ** p<.01, * p<.05, NS= not significant, DO =Domineering, VI = Vindictive, CO = Cold, SI = Socially inhibited, NO = Nonassertive, OA = Overly accommodating, SS = Self-sacrificing, IN = Intrusive/ needy
